# Autophagy in tuberculosis: advances, translational gaps, and the promise of host-directed therapies

**DOI:** 10.3389/fcimb.2026.1896798

**Published:** 2026-08-24

**Authors:** Manjunath Challa, Jessy Padma B, Sivakumar Shanmugam

**Affiliations:** Department of Bacteriology, National Institute for Research in Tuberculosis – ICMR, Chennai, India

**Keywords:** autophagy, host-directed therapies, *Mycobaterium tuberculosis*, TB treatment, tuberculosis

## Abstract

Tuberculosis (TB) is still a major global health threat, worsened by the advancement of multidrug-resistant and extensively drug-resistant strains of *Mycobacterium tuberculosis* (*Mtb*). Autophagy is a natural degradation and immune process that plays a critical role in the defence against intracellular pathogens, including *Mtb*. However, *Mtb* has developed complex mechanisms to evade autophagy by modulating phagosome-lysosome fusion, inhibiting xenophagy, and modifying host signaling pathways, including mTORC1 and AMPK, thereby suppressing epigenetically autophagy-related genes. These immune evasion strategies identify autophagy as a highly attractive target for host-directed therapies (HDTs). In many experimental models, several pharmacological and repurposed compounds, such as mTOR inhibitors, AMPK activators, lysosomal modulators, peptides, and small-molecule inhibitors, are promising factors in restoring autophagy, promoting bacterial clearance, and ameliorating inflammation. Despite the bright prospects of these preclinical studies, the intricacies of cellular pathways, the lack of selective autophagy modulators, the limited biomarkers, and the still inadequate models for translational research remain problematic. In this review, we summarise current insights into *Mtb*-mediated disruption of autophagy, evaluate emerging autophagy-enhancing HDTs, and highlight key biological and translational gaps. Further development of selective, system-level autophagy modulators may offer powerful adjunct therapies to improve TB treatment outcomes, especially in the era of escalating drug resistance.

## 1 Introduction

Tuberculosis (TB), an airborne disease caused by *Mycobacterium tuberculosis* (*Mtb*), continues to pose a danger to world health, with over 10 million cases and ranking as the second most common infectious agent-related cause of death in 2022 (WHO, 2023). One-third of the global population is estimated to carry latent tuberculosis infection (LTBI), with the risk of developing active TB at some point during their lifetime. Drug-resistant tuberculosis (DR-TB) is a significant threat to public health worldwide, making treatment more difficult and dangerous for patients and healthcare systems across the globe. In nations such as India, where the burden is particularly high, DR-TB management needs improved treatment regimens, increased public and commercial sector involvement in healthcare, and improved diagnostic capabilities (Husain et al., 2021). The occurrence of multidrug-resistant tuberculosis (MDR-TB) presents formidable challenges to treatment in the form of expensive and protracted therapies (Gina et al., 2019). These costs accentuate the considerable social, physiological, and economic hurdles to effective control of TB. More recently approved drugs, such as delamanid and bedaquiline, have higher efficacy against drug-resistant TB, whereas repurposed agents, such as linezolid, are less effective (Palucci and Delogu, 2018). This depicts the desperate need for new therapies to treat both drug-resistant and drug-susceptible tuberculosis, with host-directed therapies (HDTs) working well as additions to antibiotics.

Although antimicrobials remain essential for treating *Mtb*, host-directed therapy (HDT) is an invaluable alternative. HDT uses FDA-approved drugs to boost immune responses, facilitating bacterial clearance at lower drug doses and minimizing resistance (Gina et al., 2019). It particularly targets autophagy, a cellular degradation mechanism. Although autophagy plays a key role in the immune response against TB, *Mtb* has evolved mechanisms to bypass it (Khan and Jagannath, 2017). Hence, HDT, targeting autophagy, is an optimistic avenue for new TB treatments. Understanding *Mtb’s* interactions with the host immune system is key to designing effective vaccines and treatments. Although specific autophagy-targeting drugs do not exist at present, searching for compounds that activate autophagy might be a viable strategy for developing host-directed tuberculosis (TB) treatments that may strengthen immune defense against *Mtb* (Kim et al., 2019).

HDT targets host factors to suppress pathogen replication, enhance the immune response, and reduce inflammation, providing a potential therapy against both drug-sensitive and drug-resistant strains (Mohan and Bhattacharya, 2021). HDTs using atorvastatin, vitamin D, and metformin have been previously indicated to be potentially beneficial in favoring the immune system as well as suppressing bacterial survival, and several are currently under clinical trial (Lorente-Torres et al., 2024). It is important to understand how drugs relate to *Mtb* pathogenesis to select appropriate host-directed therapies. These therapies enhance the host’s ability to combat the infection. The role of autophagy in TB pathogenesis, escape mechanisms employed by *Mtb*, its targetability for host-directed therapy (HDT), and important autophagy enhancers in TB infection (**Table 1**) will be discussed in this review. It will also assess preclinical and clinical evidence, with emphasis on translational gaps, and conclude with some future directions for autophagy-based HDT approaches. As part of the literature search, HDT compounds related to autophagy and TB were only included.

**Table 1:** Summary of principal host-directed autophagy-modulating agents against *Mycobacterium tuberculosis*, stratified by mechanism of action and translational development stage.

Agent	Mechanism of autophagy modulation	Development stage	Key limitations
Rapamycin	mTORC1 inhibition (partial, via FKBP12); relieves ULK1 suppression, restores autophagic flux	Preclinical (*in vitro* + murine); combination studies with clofazimine	Immunosuppressive at systemic doses; narrow therapeutic window; mTORC1 inhibition can paradoxically favour *Mtb* growth in some contexts; no TB clinical trial data
Everolimus	mTORC1/mTORC2 inhibition; reduces lipid-body accumulation in infected macrophages	Preclinical (cell lines, ex vivo granulomas); repurposed from oncology/transplant use	Dose-dependent effects on mTORC2 confound autophagy-specific interpretation; immunosuppression risk in TB patients; no dedicated TB trial
Metformin (AMPK activator)	AMPK activation → ULK1/BECN1 phosphorylation; ROS generation; phagosome-lysosome fusion	Most advanced candidate – retrospective clinical cohorts and adjunctive clinical trials (largely in diabetic TB patients)	Confounded by pre-existing diabetes in most human data; risk of lactic acidosis; effect size in non-diabetic TB unclear; mechanism *in vivo* not fully autophagy-specific
Resveratrol	AMPK activation, SIRT1 activation, mTOR inhibition via IP3R-Ca^2+^ signalling	Preclinical only (cell culture)	Poor oral bioavailability/pharmacokinetics; no *in vivo* TB efficacy data; concentration-dependent pro- vs anti-proliferative effects
Vitamin D3	Cathelicidin (LL-37) induction; CaMMK-β/AMPK signalling; upregulates ATG5/BECN1	Clinical trials completed (adjunctive), efficacy inconsistent	Efficacy modified by baseline vitamin D status, VDR polymorphisms, and interaction with rifampicin/isoniazid metabolism; heterogeneous trial outcomes
Tat-BECN1 peptide (TB-1)	Disrupts BECN1 sequestration (via GLIPR2), mTORC1-independent autophagy induction	Preclinical (cell and non-TB disease models); not yet tested in TB *in vivo* models	Peptide delivery/stability challenges; no TB-specific efficacy or safety data; cell-penetrating peptide toxicity unknown in TB context
Carbamazepine	Lowers cytosolic myo-inositol/PIP2/IP3, mTOR-independent autophagy induction	Repurposed anticonvulsant; murine TB efficacy shown	Narrow therapeutic index, known hepatotoxicity and drug-drug interactions with rifampicin (CYP3A4 induction); long-term safety in TB unclear
Trehalose/disaccharides	mTOR-independent induction via lysosomal membrane permeabilisation, calcineurin-TFEB activation	Preclinical (*in vitro*; limited *in vivo* non-TB data)	High doses required; systemic delivery and metabolism poorly characterised in TB models; no clinical data
Loperamide	Autophagy induction, enhanced p62 degradation, reduced TNF-α	Preclinical (macrophage infection models)	CNS/opioid-receptor-related side effects at doses beyond antidiarrheal use; TB dosing safety not established
Src-kinase inhibitors	Modulate autophagy-linked signalling to restrict MDR/XDR *Mtb* survival	Preclinical	Class-wide off-target kinase inhibition; toxicity profile in TB patients not established
Isoniazid/Pyrazinamide (autophagy-inducing effect)	ROS generation and autophagy induction as an ancillary mechanism of established antibiotics	Clinical standard-of-care (autophagy role identified retrospectively)	Autophagy contribution to overall efficacy not isolated from direct antimycobacterial action; not a distinct HDT

For each agent, the mechanism of autophagy modulation, current stage of preclinical or clinical development, and key limitations to translational use are summarised. Agents are drawn from the compound classes discussed in Section 4 (small molecules, immunosuppressants, immunomodulators, antidiabetic and antiseizure drugs, disaccharides, peptide-based inducers, kinase inhibitors, and antibiotics). Development stage reflects the most advanced evidence reported to date, ranging from in vitro/preclinical to completed adjunctive clinical trials. A comprehensive listing of all autophagy-modulating agents discussed in this review, including full mechanistic detail, model systems, and individual literature references, is provided in **Supplementary Table 1**. ATG5/ATG12, autophagy-related proteins 5/12; AMPK, AMP-activated protein kinase; BECN1, Beclin 1; CaMKKβ, Ca^2+^/calmodulin-dependent protein kinase kinase β; CYP3A4, cytochrome P450 3A4; FKBP12, FK506-binding protein 12; IP3R, inositol 1,4,5-trisphosphate receptor; LC3, microtubule-associated protein 1 light chain 3; MDR/XDR, multidrug-resistant/extensively drug-resistant; mTORC1/2, mechanistic target of rapamycin complex 1/2; PIP2/IP3, phosphatidylinositol 4,5-bisphosphate/inositol 1,4,5-trisphosphate; ROS, reactive oxygen species; SIRT1, sirtuin 1; TFEB, transcription factor EB; TNF-α, tumor necrosis factor-alpha; ULK1, Unc-51-like autophagy activating kinase 1; VDR, vitamin D receptor.

Several reviews have previously addressed autophagy as a host-directed target in TB. Paik et al. (2019) and Silwal et al. (2018) provided detailed mechanistic accounts of AMPK/mTOR signaling and autophagy-targeted antimicrobial pathways, while our group’s earlier work Adikesavalu et al. (2021), catalogued autophagy-activating compounds and the chemical-screening platforms used to identify them. Most recently, Lei et al. (2026) offered a broad systematic account of autophagy types and *Mtb* interactions. The present review differs from these in three respects. First, rather than organising the literature primarily around signaling mechanisms or screening methodologies, we structure the evidence around translational readiness, explicitly stratifying host-directed compounds by developmental stage (preclinical, repurposed/clinical, and clinically tested) and linking this stratification to candidate biomarkers that could support clinical decision-making (**Supplementary Table 1**). Second, we include a dedicated critical appraisal (Section 6.2) of systemic blind spots in the current evidence base — in particular, the near-exclusive reliance on immortalised cell lines and murine models, and the neglect of clinical *Mtb* strain heterogeneity — that has not been a focus of prior reviews on this topic. Third, we extend the discussion beyond established pharmacology to emerging technological platforms (single-cell transcriptomics, organoid and organ-on-chip models, CRISPR-based functional screening, systems biology, and AI-assisted drug discovery) that are likely to determine whether autophagy-targeted HDTs can be rationally advanced in the next decade. Taken together, these elements position this review as a translationally oriented complement to, rather than a restatement of, the existing mechanistic literature.

## 2 Literature search strategy

A narrative literature search was conducted using PubMed/MEDLINE, Scopus, Google Scholar, Connected Papers, and Z-Library for articles published between January 2010 and June 2026. Search terms combined “autophagy” OR “xenophagy” OR “LC3-associated phagocytosis” with “tuberculosis” OR “*Mycobacterium tuberculosis*”, further combined with “host-directed therapy”, “autophagy modulator”, “mTOR”, “AMPK”, “biomarker”, or specific compound names (e.g., “rapamycin”, “metformin”, “vitamin D”). Articles were included if they reported original preclinical or clinical data or systematic/narrative review content on autophagy modulation in the context of *Mtb* infection or host-directed TB therapy and were published in English in peer-reviewed journals. Studies addressing autophagy in non-mycobacterial infections were excluded unless mechanistically foundational (e.g., core autophagy machinery) and cited for general background. Reference lists of key reviews were hand-searched to identify additional primary studies. Compounds discussed in Section 4 were restricted to those with reported activity on autophagy pathways specifically in *Mtb*-infected host cells or TB animal models, consistent with the translational focus of this review.

## 3 Autophagy: mechanism and immunological role

### 3.1 Overview of the autophagy pathway

Autophagy is a natural process where cytoplasmic contents are directed to lysosomes for degradation. Cellular homeostasis, immune function through modulation of innate and adaptive immunity, inflammatory regulation, and intracellular pathogen killing rely on this pathway (Bueno et al., 2022). Macroautophagic (sequestration of large structures into autophagosomes), chaperone-mediated autophagy (selective protein degradation), and micro autophagy (direct phagocytosis by lysosomes) all serve to regulate cellular homeostasis (Parzych and Klionsky, 2014). Macrophages can harbor *Mtb*, enabling them to evade immune defenses and survive in the body (Knowles et al., 2021). Macroautophagy, commonly called autophagy, is a cellular process that degrades damaged organelles, protein aggregates, and pathogens using lysosomes, helping maintain cellular balance. It acts upon about 30 autophagy-related proteins (Atgs) within coordinated units and is tightly regulated by numerous mechanisms (Knowles et al., 2021). When autophagy begins, Atgs, Unc-51 Like Autophagy Activating Kinase 1 (ULK1), and Beclin 1 (BECN1) initiate membrane formation around the cargo, creating autophagosomes. Through its removal of infection and regulation of inflammation, particularly in macrophages, it plays an essential role in immunity (Bah and Vergne, 2017).

Macrophages combat bacteria by engulfing them and producing reactive oxygen species (ROS) and nitric oxide (NO). However, *Mtb* evade these defenses by escaping the phagosome and replicating in the cytosol (Knowles et al., 2021). It does so by inhibiting signaling pathways and blocking the fusion of autophagosomes with lysosomes, which is necessary for degradation. Understanding autophagy in infection is key to designing novel approaches to combat these infections. *Mtb* induces autophagy in macrophages as an important defense mechanism, stimulating the maturation of autophagic lysosomes, which increase acidification and maturation of Mycobacterium-containing phagosomes, thus constraining intracellular *Mtb* survival. Autophagy’s regulatory role during pathogen infections is characterized by a bidirectional interaction, wherein microbial pathogens affect the host’s defense strategies, with effects varying by cell type, pathogen traits, and microenvironment (Zheng et al., 2022).

Increasing host control of *Mtb* infection by modulating autophagy, a key element of immune defense, offers a strategic approach to enhance TB treatment. The complex regulation of autophagy in immune processes also warrants further study, as it could inform the development of new treatment strategies.

### 3.2 Autophagy in innate and adaptive immunity

Most studies have focused on the crucial function of autophagy in immunity, as it occurs in a wide range of immune cells, including killer cells, neutrophils, and lymphocytes (He et al., 2019). It is employed by immune cells to monitor internal conditions and detect pathogens. In innate immunity, it modulates inflammation through inflammasomes, pattern recognition receptors (PRRs), and the clearance of dead cells; in adaptive immunity, it is essential for antigen presentation, thymocyte selection, and lymphocyte survival (Liu et al., 2013). For *Mtb*, activation is induced when PRRs detect bacterial elements, such as lipoproteins, lipoarabinomannan, and other pathogen-associated molecular patterns (PAMPs).

Based on research performed using animal models, it is shown that *Mtb* infection is minimized by TLR2, which senses *Mtb* lipoproteins and lipoglycans, and TLR9, which senses unmethylated CpG DNA (Ravesloot-Chávez et al., 2021). Innate immunity plays a vital role the initial response upon *Mtb* infection since it determines whether the infection is cleared or becomes active or latent TB (Tsolaki et al., 2021) There is a rising curiosity on how autophagy modulates inflammation and influences *Mtb*. Yet, autophagy plays diverse roles in infection regulation because, depending on the case, it might amplify inflammatory responses like cytokine storms (Cui et al., 2019).

### 3.3 Xenophagy and LC3-associated phagocytosis

During *Mtb* infection, neutrophils-derived extracellular vesicles (EVs) with TLR-2/6 ligands induce autophagy in macrophages, thus limiting intracellular survival of mycobacteria (Germic et al., 2019) Recently, LC-3 associated phagocytosis (LAP) has been described with unique molecular requirements that are different from those of conventional autophagy (Germic et al., 2019) Two different autophagy-related processes - i) LC3 associated phagocytosis (LAP) and ii) Xenophagy - are used by innate immune phagocytes to promote microbial digestion in lysosomes (Upadhyay and Philips, 2019) These pathways are essential for efficient microbial clearance. In macrophages, xenophagy is primarily studied in the context of *Mtb* infection (Bernard et al., 2020). Investigating unique autophagy-related processes could result in specific therapies that improve the treatment efficacy of TB infection.

## 4 Autophagy and *Mycobaterium tuberculosis*

### 4.1 Role of autophagy in TB pathogenesis

**Figure 1:**
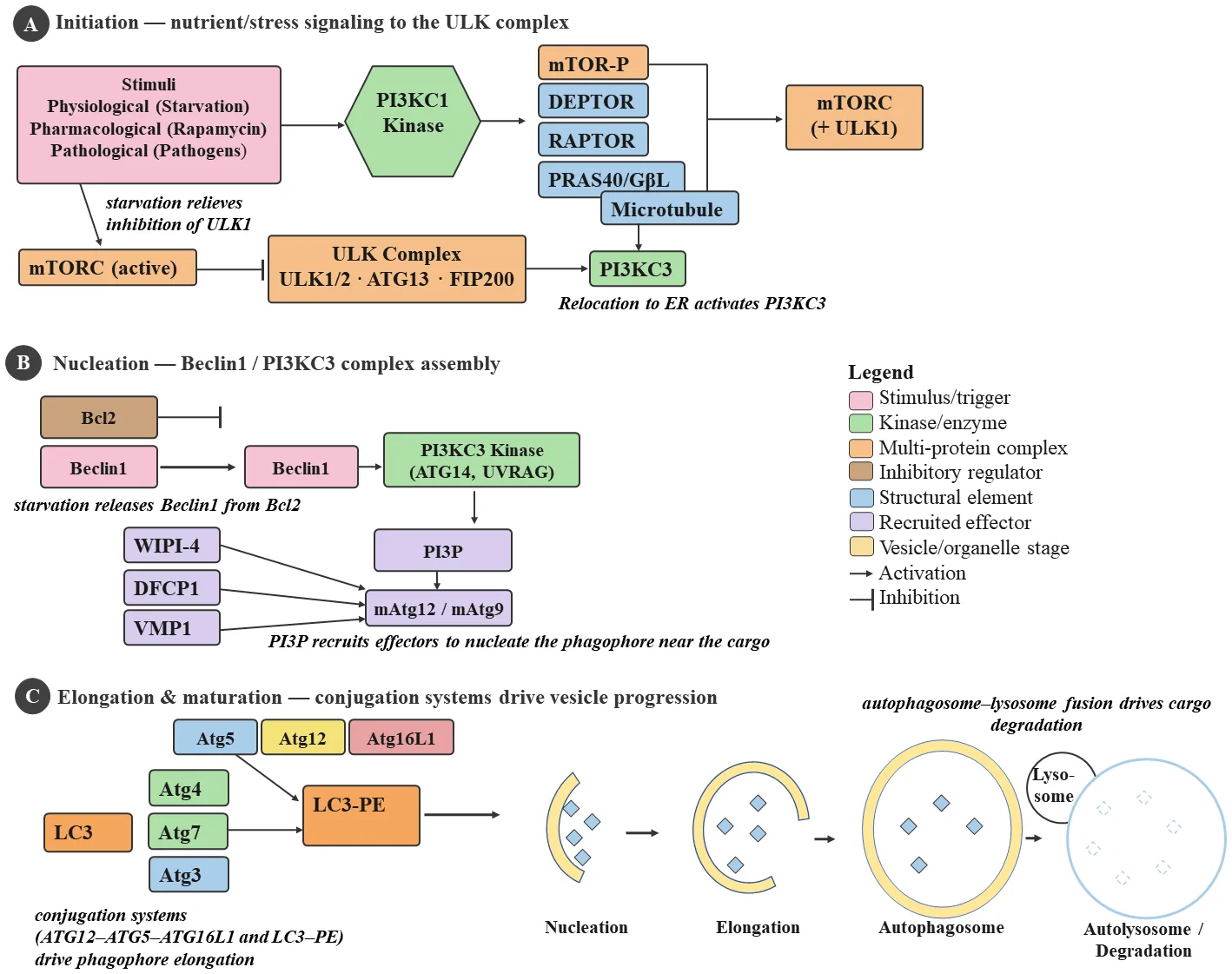
Mechanism of autophagy in mammalian cells such as the macrophage, the principal host cell for *Mtb*. **(A)** Autophagy begins with the formation of the phagophore, an initial membrane structure that originates from the endoplasmic reticulum (ER) or other intracellular membranes. This process is triggered by various stimuli, including physiological (starvation), pharmacological (rapamycin), and pathological (pathogen infection) signals. Under nutrient-rich conditions, Class I phosphatidylinositol 3-kinase (PI3KC1) activates mechanistic target of rapamycin (mTOR) as part of the mTOR complex 1 (mTORC1), which suppresses autophagy by inhibiting Unc-51-like autophagy activating kinase 1 (ULK1). During starvation or stress, mTOR activity is suppressed, allowing the ULK1 complex to activate and, in turn, stimulate the Class III phosphatidylinositol 3-kinase (PI3KC3) complex, promoting its relocation from microtubules to the ER. **(B)** Starvation also disrupts the inhibitory interaction between Beclin 1 (BECN1) and the anti-apoptotic protein Bcl-2. BECN1 increases PI3KC3 kinase activity in conjunction with UV radiation resistance-associated gene protein (UVRAG) and autophagy-related protein 14 (ATG14). The activated PI3KC3 complex produces phosphatidylinositol 3-phosphate (PI3P), which recruits essential proteins — including WD-repeat protein interacting with phosphoinositides 4 (WIPI-4), double FYVE-containing protein 1 (DFCP1), vacuole membrane protein 1 (VMP1), mammalian Atg12 (mAtg12), and the transmembrane protein mammalian Atg9 (mAtg9) — to initiate phagophore nucleation near the cargo. Phagophore elongation and membrane expansion are coordinated by two ubiquitin-like conjugation systems: the Atg5–Atg12–Atg16L1 complex and the LC3–phosphatidylethanolamine (LC3-PE) conjugation system. Atg7 and Atg10 (E1- and E2-like enzymes, respectively) facilitate Atg12 conjugation to Atg5, which then associates with Atg16L1 to form a stable complex; conjugation of LC3 to PE is enabled by cleavage of LC3 by Atg4 and subsequent processing by Atg7 and Atg3. **(C)** This conjugation drives the formation of the autophagosome. After closure, LC3-PE is removed from the outer membrane by Atg4, while LC3 remains on the inner membrane until fusion with the lysosome. The mature autophagosome fuses directly with the lysosome, exposing its contents to lysosomal enzymes for degradation; alternatively, it may first fuse with a multivesicular body (MVB) to form an amphisome before merging with the lysosome. MVBs are part of the endosome–lysosome pathway responsible for recycling membrane receptors, and their formation is tightly regulated by the Endosomal Sorting Complex Required for Transport (ESCRT) machinery; MVBs can fuse directly with either lysosomes or autophagosomes, linking endocytosis and autophagy. Collectively, these coordinated steps convert a nutrient- or stress-derived signal into a mature, cargo-laden autophagosome capable of fusing with the lysosome for degradation — the terminal event underlying autophagy’s capacity to clear intracellular pathogens, including *Mtb*. (Endoplasmic reticulum image created using BioRender).

As outlined in Section 1.1, ULK1 and BECN1 initiate autophagosome formation, which matures into an autolysosome responsible for pathogen degradation (**Figure 1**) (Bah and Vergne, 2017). In order not to be detected, *Mtb* employs various strategies in response to autophagy-related immune clearance from the host. Based on current studies, *Mtb* induces intracellular growth *in vivo* and *in vitro* by preventing recruitment of NADPH oxidase to the phagosome, thereby restricting LC3-associated phagocytosis (LAP) (Bah and Vergne, 2017).

### 4.2 Mechanisms of *Mtb*-mediated autophagy evasion

**Figure 2:**
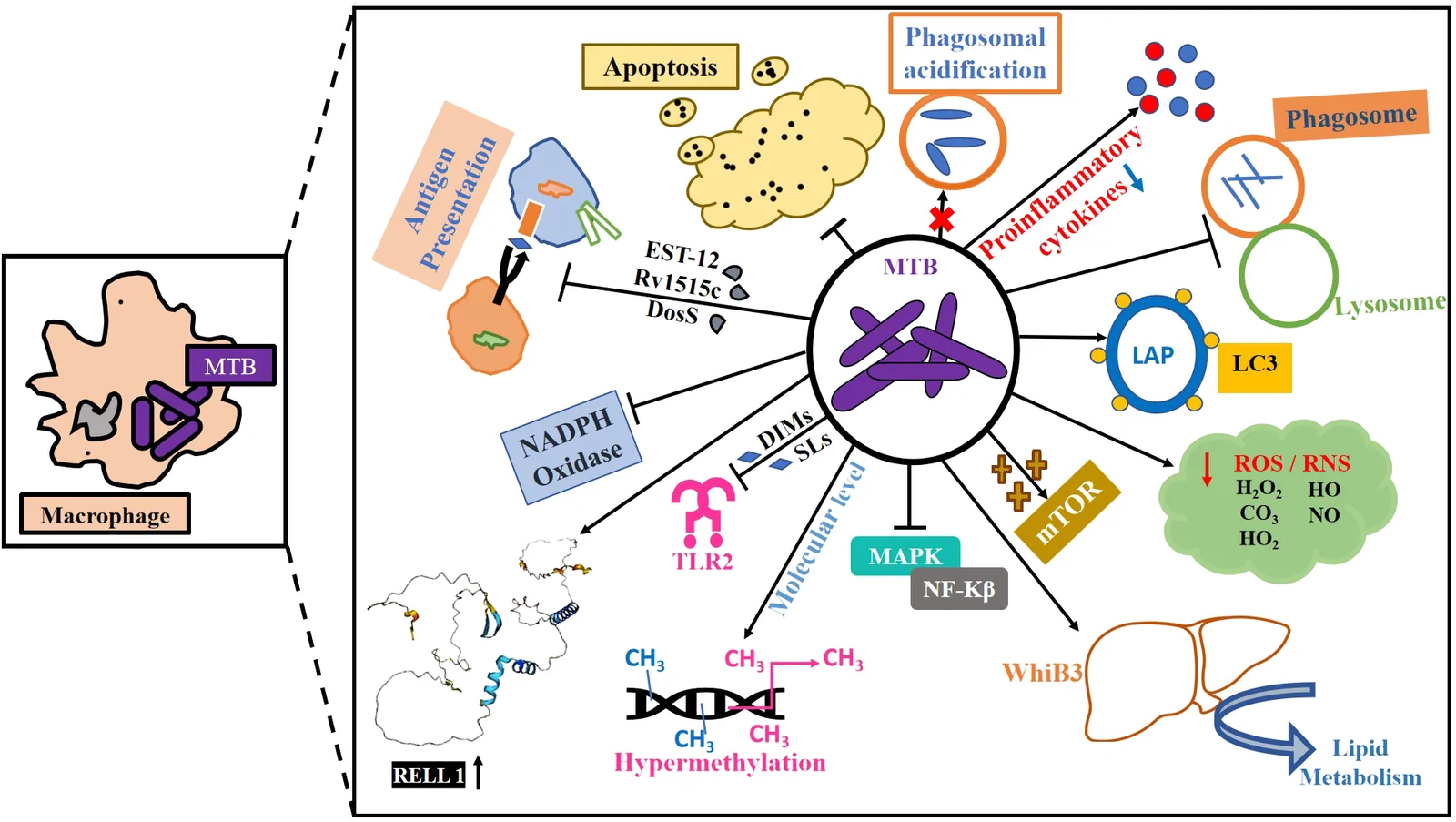
Mechanism of autophagy evasion by *Mycobacterium tuberculosis* within the macrophage. *Mtb* avoids host immune clearance by inhibiting phagosome–lysosome fusion, preventing phagosomal acidification, and suppressing pro-inflammatory cytokine responses; it also inhibits apoptosis and NADPH oxidase activity. *Mtb* further inhibits antigen presentation via effectors including EST-12, the gene product of the *Mtb* locus Rv1515c, and DosS, a sensor histidine kinase of the DosR/DosS/DosT dormancy-regulon two-component system. The *Mtb* virulence lipids phthiocerol dimycocerosates (DIMs) and sulfoglycolipids (SLs) inhibit Toll-like receptor 2 (TLR2) signaling, while the redox-sensing transcriptional regulator WhiB3 enables entry into a dormant state and the development of antibiotic tolerance. *Mtb* additionally inhibits the mitogen-activated protein kinase (MAPK) and nuclear factor-κB (NF-Kβ) pathways, reducing production of reactive oxygen and reactive nitrogen species (ROS/RNS). At the molecular level, *Mtb* suppresses autophagy through hypermethylation of histone H3 at the *Atg5* and *Atg7* gene promoters. Collectively, these evasion strategies allow *Mtb* to blunt both the autophagic and broader innate immune response of the macrophage, enabling intracellular persistence and providing the biological rationale for host-directed therapies aimed at restoring autophagic clearance.

To evade host immune responses, *Mtb* employs advanced mechanisms, including inhibition of phagosome-lysosome fusion, evasion of phagosomal acidification, and attenuation of pro-inflammatory cytokine production. To evade autophagic killing, *Mtb* inhibits host cell autophagy by manipulating host cell functions (Singh et al., 2012) (**Figure 2**). The pathogen interferes with autophagy by specifically inhibiting autophagolysosomal fusion, thereby impairing macrophage destruction of pathogens (Ebrahimifard et al., 2022). The pathogenicity of *Mtb* relies on phagosome lysis. However, autophagy is also induced by it, and the bacteria must escape it to survive within cells. Depending on the strains virulence, mycobacterial infections either lead to mTOR activation that inhibits autophagy or stimulate autophagy induction (Zullo and Lee, 2012) A growing body of evidence reveals the specific protein and lipid effectors employed by *Mtb* to disrupt distinct host lysosomal trafficking pathways (Augenstreich and Briken, 2020) In order to avoid oxidative stress and interfere with LC3-associated phagocytosis, a vital mechanism for delivering pathogens to lysosomes for destruction, *Mtb* efficiently inhibits NADPH oxidase (Chandra et al., 2022) It controls host immune responses by inhibiting apoptosis and initiating multiple cell death pathways, as reported in a recent study. Through the reduction of ROS and RNS production, inhibition of phagolysosome maturation, and inhibition of antigen presentation by effectors such as EST12, Rv1515c, and DosS, virulent strains weaken macrophage defenses (Bo et al., 2023) (**Figure 2**).

Phthiocerol dimycocerosates (DIMs) and sulfoglycolipids (SLs), key *Mtb* lipid virulence factors, interfere with autophagy by inhibiting TLR2-mediated signaling and inducing phagosomal damage, with DIMs also inhibiting acidification of LC3-positive compartments, thus ensuring bacterial survival (Bah et al., 2020) (**Figure 2**) Expanding on earlier findings, it has been established that *Mtb* employs the WhiB3 transcription factor to orchestrate lipid metabolism to allow for increased entry into a dormant state and antibiotic resistance development (Barrientos et al., 2022) Recent research on PE_PGRS41 has indicated that it facilitates bacterial persistence in macrophages by inhibiting pro-inflammatory cytokines and apoptosis and autophagy (Deng et al., 2017). Through the SecA2 pathway, which secretes effectors SapM (phosphatase) and PknG (kinase), these prevent phagosome maturation and increase bacterial survival, eluding macrophage defenses (Zulauf et al., 2018).

microRNA-25 (miR-25) promotes *Mtb* survival within host cells by targeting Niemann-Pick type C1 (NPC1), thereby disrupting autophagic flux and inhibiting autophagosome-lysosome fusion, and this regulation occurs through the NFκB pathway (Dong et al., 2022) (Zulauf et al., 2018). K63-linked ubiquitination of PPE68 by MKRN1 facilitates its interaction with SHP1 and TRAF6, thereby suppressing NF-κB and MAPK activation and enhancing *Mtb* survival in macrophages (Dou et al., 2022). Earlier research has shown that RELL1 (Receptor expressed in lymphoid tissues-like protein 1) overexpression suppresses autophagy, thus enhancing the survival of *Mtb* in macrophages (Feng et al., 2020). NapM, an *Mtb* stress-inducible nucleoid-associated protein, suppresses DnaA, thereby inhibiting DNA replication initiation and enabling bacterial survival under unfavorable conditions. The mechanism enables *Mtb* to counteract autophagy in macrophages (Liu et al., 2019). At the molecular level, Mtb suppresses autophagy through dual epigenetic mechanisms: it induces hypermethylation of histone H3K9 and H3K27 at the ATG5 and ATG7 promoters, and reduces acetylation by upregulating HDAC3, thereby facilitating intracellular persistence (Sengupta et al., 2021) (**Figure 2**).

### 4.3 Host-directed therapeutics targeting autophagy

To treat infections such as TB, host-directed drugs can modulate autophagy, a self-degradative process that maintains cellular homeostasis (Verma et al., 2021). With the emergence of drug resistance, toxicity, and long-term therapy, TB remains a global health challenge. *Mtb* can manipulate autophagic flux so that the induction of autophagy inhibits *Mtb* growth. At the same time, its blockade promotes bacterial growth — indicative of an integrated component of the host defense mechanism against *Mtb* infection. Interestingly, *Mtb* is known to co-localize with essential autophagy markers such as ATG5, Beclin-1 (BECN1), and LC3 (Pahari et al., 2020). Host-directed therapy offers a promising strategy to improve treatment efficacy for TB, especially against multi-drug-resistant (MDR) and extensively drug-resistant strains (XDR) by targeting host immune pathways to enhance both protective and pathological responses. For instance, one study demonstrated that Vitamin D3 enhances innate immune responses against *Mtb* by inducing autophagy through cathelicidin (Yuk et al., 2009).

Separately, another study highlights NR1D1, an orphan nuclear receptor that enhances the antimicrobial activity of human macrophages, as a modulator of autophagy (Chandra et al., 2015). The key role of peroxisome proliferator-activated receptor alpha (PPAR-α) is to enhance immune responses against *Mtb* through various pathways, including autophagy, lysosomal biogenesis, and phagosomal maturation (Kim et al., 2017). Additional investigations indicate that deficiencies in Estrogen-related receptor alpha (ERRα) result in impaired phagosomal maturation and a reduced antimicrobial response, underscoring its role in the development of host-directed therapeutics (Kim et al., 2018).

Antimycobacterial agents, such as isoniazid (INH) and pyrazinamide (PZA), induce autophagy and reactive oxygen species (ROS) generation in host cells, which are critical processes for controlling *Mtb* infection (Kim et al., 2012). Treated with thiostrepton, studies show that endoplasmic reticulum (ER) stress-mediated autophagy is activated in host cells, enhancing the clearance of intracellular pathogens (Zheng et al., 2015). In addition, ambroxol was found to induce autophagy both *in vitro* and *in vivo* at clinically relevant doses, thereby facilitating the demise of mycobacteria within macrophages (Choi et al., 2018). To address drug-resistant *Mtb*, a 5-nitro-1,10-phenanthroline compound was found to kill bacteria by inducing autophagy in macrophages (Kidwai et al., 2017).

Interestingly, Loperamide exhibits a promising adjunctive treatment for TB by inducing autophagy, enhancing p62 degradation, and inhibiting *Mtb* growth in macrophages and decreasing TNF-α levels (Juárez et al., 2016). Also, Carbamazepine (CBZ) induces autophagic clearance of *Mtb* in macrophages and decreases bacterial burden and inflammation in mice, effects not attained by conventional TB drugs (Guler et al., 2021). Treatment for resistant TB is expanded by HDT, which uses Src kinase inhibitors to effectively reduce the survival of XDR and MDR strains of *Mtb* (Paik et al., 2019). In fact, HDTs can reduce infection and disease activation by strengthening immune responses to improve defense against TB, particularly in high-risk and latent infections (Young et al., 2020). Nevertheless, the full range of effects resulting from these perturbations remains challenging to define and thus requires further clarification to develop improved therapeutic approaches for pathogenic mycobacteria.

#### 4.3.1 Enhancing autophagy to reduce bacterial load and inflammation

Autophagy controls immune cell function and viability, important in modulating inflammation and pathogen elimination in the context of TB infection (Qian et al., 2017). Autophagy modulates immune responses by controlling PAMP- and DAMP- triggered inflammasome activation, for example, NLRP3, by removing inflammatory mediators such as ROS, mtDNA, and LPS, limiting excessive inflammation but contributing to host defense (Cadwell, 2016). Autophagy receptors such as p62, OPTN, and NDP52 mark inflammatory targets, such as pathogens and dysfunctional organelles, for degradation, eliminating inflammation and aiding in immune function by mechanisms such as xenophagy and mitophagy (Deretic, 2021).

It inhibits excessive inflammation by degrading components of the inflammasome, such as PYCARD, and by clearing damaged mitochondria, thereby releasing pro-inflammatory signals such as mtDNA and ROS (Brady et al., 2018). *In vivo* studies have demonstrated that enhancing autophagy limits *Mtb* growth while reducing inflammation-induced tissue damage (Castillo et al., 2012). However, autophagy depends on galectins and regulatory proteins such as PRKN and OPTN, which can mediate the protective response to cellular damage, suggesting potential pharmacological targets for chronic inflammatory diseases (Deretic and Levine, 2018). A recent study demonstrated that a pan-FGFR inhibitor, LY2874455, induces p62-dependent autophagy and degradation of the immunoproteasome, leading to suppression of NF-Kβ- dependent inflammation and potential therapeutic potential for inflammatory diseases (Zhou et al., 2024). Autophagy induction during infectious diseases has therapeutic importance, as it not only promotes xenophagy but also helps reduce hyperinflammation and tissue damage (Matsuzawa-Ishimoto et al., 2018). Other FDA-approved drugs, such as tamoxifen, Ridaifen-B, and gefitinib, help the host facilitate autophagy, which can assist TB treatment and augment the BCG vaccine (Khan and Jagannath, 2017).

### 4.4 Pharmacological activators of autophagy

Autophagy plays two key roles in helping cells survive, and its impact depends on the cell type and the type of stress it faces. Pharmacological regulation of autophagy is a highly promising therapeutic approach for many diseases, including cancer, neurodegeneration, and cardiovascular disease. Autophagy modulators can stimulate or inhibit autophagic activity, and their mechanisms of action are complex and multifaceted. They affect cells through several processes and can have various impacts on cell function, which is important to understand when evaluating how well these drugs work and what side effects they might cause.

#### 4.4.1 mTOR inhibitors

mTOR is a serine/threonine kinase responsible for coordinating several important cellular processes, such as energy metabolism, protein synthesis, and regulation of programmed cell death (Hung et al., 2012). It operates through two complexes, mTORC1 and mTORC2, which differ in composition and rapamycin sensitivity. mTORC1, containing RAPTOR, is highly responsive to rapamycin, unlike mTORC2, which remains less studied (Ben-Sahra and Manning, 2017) — a distinction relevant to the pharmacology discussed below, since rapamycin and everolimus differ in their mTORC1/mTORC2 selectivity (see 4.1.1–4.1.2). As outlined in Section 1.1, mTORC1 inhibits the ULK1 complex to suppress autophagy; *Mtb* exploits this by using the PI3K/AKT pathway to activate mTORC1 and evade autophagic killing. This mTOR-activating strategy is not universal across *Mtb* infection: as noted in Section 2.2, the direction of the host mTOR response can be strain- and virulence-dependent, with some mycobacterial infections activating mTOR to suppress autophagy while others instead directly stimulate autophagy induction (Zullo and Lee, 2012). The mTOR-inhibitor pharmacology discussed below should therefore be interpreted against this strain-dependent baseline rather than a single uniform mechanism of *Mtb*-driven autophagy suppression. In some situations, mTORC1 inhibition can paradoxically enhance *Mtb* proliferation, necessitating targeted therapies (Patel et al., 2024). *Mtb* uses the PI3K/AKT pathway to control mTORC1 to stop autophagy and escape killing. In some situations, mTORC1 inhibition can paradoxically enhance *Mtb* proliferation, necessitating targeted therapies (Patel et al., 2024).

##### 4.4.1.1 Rapamycin

By partially inhibiting mTORC1 substrates such as 4E-BP1, rapamycin disturbs mTORC1’s negative feedback control on PI3K/AKT signaling, therefore increasing Akt activation and improving cell survival (Li et al., 2014). A recent study indicated that rapamycin and clofazimine (CFZ) work well together to destroy MDR and XDR strains of *Mtb*, while CFZ enhances T-cell memory, rapamycin initiates autophagy to eliminate the infections (Singh et al., 2023). Another study demonstrated rapamycin’s capacity to reduce pulmonary inflammation and necrotic granulomas, further supporting its potential as a host-directed therapeutic option for TB (Bhatt et al., 2021). Spray-dried rapamycin-loaded particles target macrophages, enhance intracellular *Mtb* clearance, and are suited for dry powder inhalation, advancing host-directed TB therapy (Gupta et al., 2014) (**Supplementary Table 1**).

##### 4.4.1.2 Everolimus

Everolimus, a mTOR inhibitor, increases autophagy, providing promise against resistant *Mtb* strains and decreasing DOTS dependency (Cerni et al., 2019). It improves *Mtb* clearance by activating autophagy through mTORC1 inhibition, thus being a possible adjunct for the treatment of TB (Singh and Subbian, 2018). It has demonstrated pro-inflammatory TNF-α levels reduction in granulomas and improved lung function in TB patients (Amin et al., 2021). Everolimus blocks mTORC1 and mTORC2 at higher doses, reducing cell growth and phosphorylation of S6K1 and 4EBP1 (Saran et al., 2015). When combined with antibiotics like INH and PZA, it enhances effects by reducing oxidative stress and bacterial load, also lowering TNF-α levels, modulating immune responses (Raien et al., 2023) (**Supplementary Table 1**).

A recent study shows that everolimus reduces lipid body accumulation in *Mtb*-infected THP1 macrophages, improving bacterial control (Cao et al., 2021). This implies that its anti-mycobacterial effects could be caused by alterations in lipid metabolism, thereby supporting its possible use as an adjunctive TB treatment. Suggesting both autophagy-dependent and independent pathways, higher concentrations of everolimus (2nM) increased LC3B expression and lowered *Mtb* CFUs in granulomas (Ashley et al., 2020). Decreasing cyclin D1 concentrations suppresses mTORC1 and induces G1 arrest; autophagy and proteasomal pathways play critical roles in degradation (Chen et al., 2019). Through the promotion of bacterial elimination, optimization of immune cell function, and disruption of the survival mechanisms of intracellular pathogens, everolimus induces G1 cell cycle arrest and autophagy, fostering HDT approaches.

Taken together, rapamycin and everolimus provide converging evidence that mTORC1 inhibition can induce autophagy and reduce *Mtb* burden, but the two effects reported for this drug class are not fully reconcilable: mTORC1 inhibition has been shown to reduce pulmonary inflammation and necrotic granuloma formation (Bhatt et al., 2021), yet in other contexts the same inhibition has been reported to enhance *Mtb* proliferation paradoxically (Patel et al., 2024). This discrepancy has not been resolved and is unlikely to reflect a simple contradiction; more probable explanations include dose-dependent effects (partial versus complete mTORC1 blockade), the timing of inhibition relative to the stage of infection, differential inhibition of mTORC1 versus mTORC2, and differences between the murine and human macrophage systems used across studies. Until these variables are controlled directly, mTOR inhibition should be regarded as a context-dependent rather than uniformly beneficial strategy for TB HDT.

#### 4.4.2 AMP-activated protein kinase activators

A serine/threonine kinase known as AMPK, regulates immunological reactions and energy expenditure in the case of infection. It enhances autophagy and macrophage reactions in mycobacterial infection, but it also enhances TB immunopathology via Matrix metalloproteinase-8 (MMP-8) (Silwal et al., 2018) — a dual, context-dependent role that parallels the mTORC1-inhibition paradox described in Section 4.1, and one that is equally important to account for when interpreting AMPK-activator pharmacology below. As outlined in Section 1.1, AMPK activates autophagy by phosphorylating ULK1 and BECN1 and relieving mTORC1 suppression; through stabilization of autophagy and AMPK proteins, IRGM links immunity to pathogen clearance and strengthens lysosomal defenses (Jo et al., 2019). Furthermore, AMPK-dependent autophagy is also activated by genotoxic and oxidative stress, highlighting its function in adaptive cellular responses (Saikia and Joseph, 2021).

Further, AMPK is also a crucial player in autophagosome - lysosome fusion by controlling lysosomal biogenesis and function through its interaction with TFEB, CARM1, and mTORC1 pathways. AMPK coordinates nutrient and energy signals to turn up autophagy (Wang et al., 2022). AMPK activation in the lysosome, mediated by the AXIN and v-ATPase regulator complex, links energy stress to autophagy regulation and downregulates mTORC1 signaling (Li and Chen, 2019). This highlights the therapeutic potential of AMPK in TB treatments.

##### 4.4.2.1 Metformin

Metformin (MET), a biguanide and Food and Drug Administration (FDA)-approved diabetes medication, act as an adjunctive tuberculosis (TB) therapy by activating adenosine monophosphate-activated protein kinase (AMPK), promoting autophagy, and enhancing *Mtb* control through generation of reactive oxygen species and fusion of phagosomes and lysosomes (Novita et al., 2021). Retrospective studies have shown that MET could benefit TB treatment; however, prospective trials need to be conducted to clarify its role, including drug interactions, and to manage potential toxicities particularly lactic acidosis (Yew et al., 2019). As an AMPK modulator, MET inhibits *Mtb* growth, enhances anti-TB drug efficacy, increases T-cell responses, and strengthens immunity (Mishra et al., 2022).

It also inhibits bacterial NDH-I, an essential respiratory complex of *Mtb*, and hence can be used to break *Mtb* persistence (Naicker et al., 2020). MET treatment in *Mtb*-infected mice alleviated inflammation and tissue injury by regulating inflammatory gene expression and reducing markers such as IL-1β, TNF-α, and IL-6, enhancing disease pathology (Singhal et al., 2014). One study showed that MET reprograms CD8+ T cells, boosting BCG vaccine efficacy and host immunity against *Mtb* (Böhme et al., 2020). Moreover, MET was found to induce autophagy and stimulate autophagic flux by upregulating LC3-II and downregulating the levels of p62, along with activating AMPK and suppressing mTOR signaling in Hepatocellular Carcinoma cells (Gao et al., 2020). Metformin also acts on pathways such as Redd1/mTOR, STAT, SIRT, and MAPK/ERK, which modulate autophagy based on tissue or disease (Lu et al., 2021). A study in 2023 demonstrated that when MET was combined with INH, TB control was improved, and potential against drug-resistant strains via macrophage survival was observed (Naicker et al., 2023). In another study, MET has shown potential in reducing the progression of latent tuberculosis to active disease, enhancing sputum conversion, and improving patient outcomes, particularly in diabetic individuals (Sutter et al., 2022).

Metformin is the most advanced clinically available AMPK-targeting agent with evidence spanning from cell and murine models to retrospective human cohorts and adjunctive trials (Naicker et al., 2023; Sutter et al., 2022). Alternatively, resveratrol has only been studied in cell culture systems to date, and there are no *Mtb* specific *in vivo* efficacy or human data available. Its reported anti-cancer and antiproliferative effects at higher concentrations make it difficult to directly extrapolate AMPK activating potential to a TB context. Thus, these two agents should not be considered evidentially equivalent AMPK activators, although they share a common proposed mechanism.

##### 4.4.2.2 Resveratrol

Resveratrol, a plant-derived polyphenol, regulates cell proliferation, differentiation, apoptosis, and autophagy, with its effects varying with concentration: low doses promote cell proliferation, whereas higher doses exhibit anticancer properties (Borriello et al., 2014). Recent findings indicate that resveratrol induces autophagy through IP3R-dependent cytosolic Ca2+ signaling, even as it depletes ER Ca2+ independently of IP3Rs (Luyten et al., 2017). Resveratrol induces autophagy by inhibiting mTOR and activating AMPK and SIRT (Tian et al., 2019). It also increases autophagic vacuoles, LC3II/I ratio and decreases p62 levels, confirming autophagy induction (Josifovska et al., 2020). In a clinical study, resveratrol was shown to block Akt signaling in cancer cells with an overactive PI3K/Akt/mTOR pathway (Berman et al., 2017).

#### 4.4.3 Lysosomal modulators

Lysosomal modulators are compounds that affect lysosomal function by either enhancing or suppressing its activity. Lysosomes are integral components in a multitude of cellular mechanisms, transcending their role as repositories for recycling. They serve as pivotal signaling centers essential for the detection of energy levels and amino acids, transduction of signals and regulation of autophagy (Perera and Zoncu, 2016; Yang and Wang, 2021).

#### 4.4.4 Peptide-based modulators

According to a recent study, Iztli peptide 1 (IP-1), a synthetic AMP, binds ATP and lowers its intracellular levels caused autophagy in HEK293T cells and macrophages. It was also successful in treating drug-sensitive and MDR-TB infected mice (Peláez Coyotl et al., 2020). In *Mtb* infections, host-directed drugs that induce autophagy and target antimicrobial peptides (STAMPs) specifically increase antimicrobial activity, decrease host toxicity, and decrease lung damage (Muciño et al., 2016). A BH3-mimetic peptide, such as ABT737, breaks the Bcl-XL/Bcl2-BECN1 complex, inducing autophagy and inhibiting apoptosis (Cecconi, 2009). Another study identified that the DNA tetrahedron-BH3 peptide nanosystem (Tdpep) induces autophagy explicitly by inhibiting BECN1-Bcl2 binding, which enhances LC3 puncta and decreases ROS levels (Hema Naveena et al., 2024).

##### 4.4.4.1 Tat-BECN1 peptide

Tat-BECN1 peptide is a potent autophagy inducer that enhances LC3 conversion and autophagosome formation (Shoji-Kawata et al., 2013). Also, in a study by Zhou et al, Tat-P helped restore autophagy and repair lung cells (Zhou et al., 2023). It has also been demonstrated that TB-1 alleviates hepatic steatosis, fibrosis, and metabolic dysfunction in MASLD by promoting autophagy (Chen et al., 2024). It initiates autophagy through BECN1 and GLIPR2, evading mTORC1 (He et al., 2022).

##### 4.4.4.2 Carbamazepine

CBZ increased the autophagy and proteasomal degradation of mutant ATZ through a TOR-independent route, according to a study on alpha1-antitrypsin (Hidvegi et al., 2010). By increasing autophagy, it eliminated toxic aggregates and decreased liver damage in alpha-1-antitrypsin deficiency (ATD) and fibrinogen storage disorder (FSD) (Puls et al., 2013). CBZ and valproic acid (VPA) promote the autophagic elimination of mycobacteria by reducing cytosolic myo-inositol, which lowers PIP2 and IP3 levels and starts autophagy (Schiebler et al., 2015).

##### 4.4.4.3 Vitamin D

Vitamin D3-induced immunity has been shown to link with autophagy, where it enhances autophagy via cathelicidin (hCAP) to combat *Mtb* (Yuk et al., 2009). *Mtb* disrupts phagosomal integrity via ESX-1 secretion system, leading to activation of TLR1 signaling and vitamin D_3,_ which induces LL-37 and autophagy through CaMMK-β/AMPK (Periyasamy et al., 2020). However, a study demonstrated that vitamin D_3_ promotes autophagy in *Mtb*-infected THP1-cells and alleviates pulmonary injury in mice, by restoring autophagic function through modulation of Ca^2+^ signaling (Wu et al., 2022) Vitamin D strengthens innate immunity by inducing antimicrobial peptides and promote bacterial killing, while, it suppresses adaptive immunity by inhibiting Th1 cytokines (Wei and Christakos, 2015) It upregulates hCAP via macrophage CYP27b1, counteracting pathogen immune evasion (Adams et al., 2009). IL-15 links TLR2/1 activation to vitamin D-dependent antimicrobial pathway in monocytes and macrophages, where it enhances CYP27b1 expression (Krutzik et al., 2008).

Vitamin D boosts antibacterial autophagy by increasing cathelicidin, BECN1, and ATG5, which helps in the fusion of phagosomes with lysosomes to eliminate *Mtb* (Bhutia, 2022). It also raises ATG5 and BECN1 through p38 MAPK and ERK1/2 signaling (Afsal and Selvaraj, 2016). 1,25D3, the active form of vitamin D, inhibits both HIV and *Mtb* replication in co-infected macrophages via CAMP-dependent autophagy requiring phagosomal maturation (Campbell and Spector, 2012). However, Vitamin D exhibits antimycobacterial effects, but its clinical role in TB remains unclear; it might enhance PZA, but its metabolism is affected by rifampicin and isoniazid. Also, Vitamin D and A show synergistic effect by binding their intracellular receptors (VDR and RAR) respectively, then interact with RXR to regulate gene expression (Anand et al., 2008; Papagni et al., 2022).

##### 4.4.4.4 Disaccharides

Several Disaccharides have been reported to induce autophagy, primarily through mTORC1-independent mechanisms. Trehalose, sucrose, and raffinose induce autophagy without inhibiting mTOR (Chen et al., 2016). Trehalose, a natural disaccharide, induces autophagy independently of mTOR and shows therapeutic potential for neurodegenerative diseases (Hosseinpour-Moghaddam et al., 2018). It actually activates transient lysosomal membrane permeabilisation (LMP), leading to calcium release and activation of calcineurin, which dephosphorylates TFEB, enhancing autophagy and lysosomal biogenesis (Rusmini et al., 2019). In a recent study, it’s seen that trehalose reduced MI-induced cardiac remodeling and dysfunction by activating autophagy and enhancing mitophagy (Sciarretta et al., 2018). Another study showed that trehalose reduces intracellular pathological Prion protein (PrPSc) via autophagy (Aguib et al., 2009). Trehalose restores autophagy via Ptdins (3,5) P2, which helps reduce *Mtb*, NTM, and HIV-1 both *in vitro* and vivo, revealing its therapeutic potential (Sharma et al., 2021). A study found that glucose induces autophagy under starvation conditions via p38 MAPK activation rather than mTOR inhibition (Moruno-Manchón et al., 2013). There is further evidence suggesting that sucrose induces autophagy by increasing LC3-II levels without toxicity, enhancing autophagic flux, and acting independently of mTOR (Higuchi et al., 2015).

Vitamin D3 illustrates a mechanism-to-clinic gap rather than a straightforward translational success: its capacity to induce cathelicidin, upregulate ATG5/BECN1, and enhance phagosome-lysosome fusion is consistently reproduced across *in vitro* macrophage studies (Yuk et al., 2009; Bhutia, 2022; Afsal and Selvaraj, 2016), yet its clinical role in TB remains unclear, with efficacy modified by baseline vitamin D status and by interaction with rifampicin and isoniazid metabolism. This inconsistency should be read as a reproducibility gap between preclinical mechanism and clinical outcome, rather than as evidence against a role for vitamin D in autophagy-based HDT.

A similar caveat applies to the mTOR-independent inducers discussed above. Disaccharides such as trehalose and peptide-based inducers such as Tat-BECN1 and the DNA tetrahedron-BH3 nanosystem (Tdpep) are mechanistically well characterised *in vitro*, but the supporting evidence for both classes derives almost entirely from non-TB disease models or *Mtb*-infected cell lines, with no reported *Mtb in vivo* efficacy or human data. These agents should not be read as occupying the same evidentiary tier as metformin or vitamin D3, which have at least reached human cohorts; at present, they represent early-stage mechanistic leads rather than clinically validated candidates.

## 5 Challenges in clinical translation

Modulating autophagy in the human body has emerged as a promising avenue for the treatment of TB and related diseases. The role of autophagy in combating TB is critical, as it bolsters the immune response and facilitates the clearance of pathogenic bacteria. Given that *Mtb* possesses mechanisms to evade autophagic degradation, a focused approach on enhancing autophagy may lead to improved outcomes in therapeutic interventions and vaccination strategies (C et al., 2011). Nevertheless, several obstacles persist that hinder the practical application of autophagy modulation in clinical treatments. The common hindrances of autophagy-targeting therapeutic approaches fall into three broad categories: regulatory obstacles, pharmacological constraints, and biological complexity. Current pharmacological agents are non-specific, and the effects of autophagy modification across various clinical situations are unpredictable. While autophagy is critical to maintaining cellular homeostasis, its impact can vary in different situations and can be both harmful and beneficial. This dual nature complicates therapeutic targeting, since depending on the cellular environment, regulation of autophagy may have different consequences (Pentimalli, 2019).

The lack of specificity of frequently used autophagy inhibitors, including chloroquine, severely limits their clinical use and complicates the assessment of therapeutic efficacy. This highlights the urgent need to develop more targeted therapeutic strategies to address these challenges effectively (Ferreira et al., 2021; Gewirtz, 2016). Its role in treatment depends on cellular context, functioning either as a cell survival mechanism or a cell death mediator, rendering treatment uncertain (Gewirtz, 2016). Drug discovery targeting autophagy relies on challenges like unstable assays, absence of specific biomarkers, and intricacy in the autophagy pathway (Kocak et al., 2022). Autophagy-inducing compounds have been trialed as adjunctive therapy for TB with mixed results (e.g. vitamin D3 supplementation trials have shown variable effects on sputum culture conversion depending on dosing regimen, vitamin D receptor genotype, and co-administered anti-tubercular drugs) (Adikesavalu et al., 2021). There are few autophagy modulators with minimal off-target effects, thus limiting their therapeutic potential; hence, the significance of more precise drug design. In addition, current drugs often act on multiple pathways, leading to unintended cellular side effects. Moreover, since there are no reliable biomarkers, it is more difficult to measure the molecular effects of autophagy-targeting drugs, which complicates therapeutic development. Therefore, the existing expertise regarding the clinical translation of autophagy induction is limited, as the majority of studies have been conducted in murine models or cell lines, as opposed to in human patients.

Although no autophagy biomarker has yet been validated for clinical use in TB, However, certain markers can be helpful in development of new autophagy-related trials. The best known and most extensively studied autophagosome marker is LC3-II (lipidated and membrane-bound form of LC3-I), which is a marker of autophagy induction, but it does not reflect the dynamics of the process as the LC3-II level is balanced by induction and degradation. Thus, to make sure there is an increase in autophagosome number, it is necessary to use specific flux assays (bafilomycin A1 and/or chloroquine blockade), since LC3-II level can increase as a result of increased induction and decreased lysosomal degradation. Another protein associated with autophagy is p62/SQSTM1, which is also a marker of autophagy as it binds with autophagic receptors and cargo, and is degraded lysosomally. Consequently, the decrease in p62 is a sign of autophagy flux; p62 levels were changed after application of several autophagy inducers (loperamide, metformin). Expression and dissociation of Beclin-1 (BECN1) protein from Bcl-2 are the possible markers of autophagy induction capacity. Other essential ATG proteins (ATG5-ATG12-ATG16L1 complex, ATG7) can potentially be examined by measuring their transcripts or proteins in the blood mononuclear cells, although this approach has not yet been systemically explored in TB populations. In addition to single proteins, host transcriptomic biomarkers – multiple gene RNA profiles in blood that have already been established to be effective in diagnosing TB and predicting responses to treatment – represent a scalable way of capturing the autophagy pathway activation as part of a more general host response signature, and can be developed concurrently with currently used TB biomarker platforms rather than as an independent test. The validation of these candidates will inevitably involve standardized flux-based measurements in longitudinal human cohorts, which has not yet been done in the context of TB host-directed therapy studies.

## 6 Future directions and research gaps

### 6.1 Towards more selective HDTs

Targeting autophagy for the treatment of TB necessitates evaluating novel approaches to improve host immune responses against *Mtb*. These approaches enhance the efficiency of current treatments and facilitate the introduction of novel pharmaceuticals by modulating autophagic pathways. Among the various approaches, the utilization of functionalised nanomaterials has been identified as a means to augment autophagic processes within macrophages, promoting antimycobacterial activity against *Mtb* (Liu et al., 2024). In a related context, anti-diabetic pharmaceuticals such as Troglitazone have been shown to activate autophagy via the LKB1-AMPK signaling pathway, demonstrating synergistic effects when combined with traditional anti-TB medications (Bi et al., 2025). However, there are still some hurdles to overcome in implementing these findings in clinical practice. Although autophagy modulators could be highly effective in treating TB, the obstacles we face indicate that we need more focused research and development to translate these ideas into effective therapies. Further studies on novel compounds and approaches provide hope for generating more selective and potent autophagy-modulating drugs. Besides autophagy, recently identified regulated cell death pathways such as PANoptosis have been proposed as potential host-directed therapeutic targets. Future studies are needed to investigate the crosstalk between autophagy and PANoptosis during *Mtb* infection and to identify combinatorial approaches to improve bacterial clearance and prevent excessive inflammation (Yao et al., 2026).

In addition to the pharmacological strategies described above, there are a few other developing technologies that may influence the future development of HDT against *Mtb* based on autophagy modulation. Single-cell transcriptomics can resolve macrophage and granuloma heterogeneity that is invisible to bulk assays, potentially identifying autophagy-competent versus autophagy-suppressed cell subpopulations within the same lesion and clarifying why systemic autophagy modulators show inconsistent efficacy. Organoids and lung-on-a-chip technologies, which more closely mimic tissue structure and human-specific immune signalling compared to immortalized cell lines and murine infection models, provide a way forward to screen candidate HDTs (such as everolimus and metformin) in systems more relevant to human granuloma physiology before the design of clinical trials. Functional screening using the CRISPR technology, including genome-scale or autophagy-centric knockout/knockdown screening of *Mtb*-infected macrophages, provides a systematic way to uncover new autophagy regulators and off-target liabilities associated with existing drugs to solve the specificity issue. A systems biology approach combining the integration of transcriptomic, proteomic, and metabolic profiling at the host-pathogen interface is required to elucidate the interrelated lipid metabolism, epigenetic, and immune reprogramming processes taking place within the host, instead of studying autophagy as an independent pathway. Lastly, AI-enabled drug discovery, including structure-based virtual screening and machine-learning-based repositioning of FDA-approved drugs, would significantly broaden the scope of the screening process for potential autophagy modulators beyond the current limited number of mTOR inhibitors and AMPK activators.

### 6.2 Uncovering the gaps: strategic blind spots in autophagy-based HDT for tuberculosis

Autophagy is a context-dependent regulator of host immunity, and its therapeutic manipulation requires precision rather than broad activation or inhibition. This will be supported by the future development of selective autophagy modulators, biomarker-driven patient stratification, and targeted delivery approaches to enable the clinical translation of autophagy-based host-directed therapies for tuberculosis (He and Qi, 2026). Even with increasing interest in pharmacological autophagy inducers and host-directed therapies against *Mtb*, the existing body of research remains fragmented and neglects the pathogen’s adaptive strategies. The bias toward studying autophagy modulators based on their potential to activate ignores how *Mtb* actively re-engineers the host response to evade autophagic clearance. By doing so, we often miss important details about how *Mtb* interacts with the host and potentially oversimplify therapeutic targets. *Mtb*’s ability to manipulate key regulators such as mTORC1, AMPK, and TFEB not only subverts drug action but also highlights the shortcomings of our model systems and experimental designs. Much of the information in the field is derived from immortalised or murine cell lines, which may not recapitulate human immunological responses or the metabolic subtleties that *Mtb* has evolved to exploit.

In addition, studies infrequently control for the metabolic or epigenetic changes introduced by *Mtb* into host cells, a bias that has significant consequences for drug discovery and translation success. As we proceed, a strategic change is needed. We need to embrace integrated platforms that study both autophagy induction and bacterial countermeasures in tandem. Host lipid metabolism, transcriptional reprogramming, and immune modulation cannot be viewed as distinct domains but as interlinked layers that *Mtb* traverses with precision. Researchers also need to pay greater attention to the heterogeneity of clinical *Mtb* strains and to their differential effects on autophagy, an under-investigated yet essential area. Lastly, although inhibiting upstream regulators, such as mTORC1, has therapeutic potential, downstream effectors, such as TFEB, could provide more targeted, less immunosuppressive options worth further investigation. If we are to bypass *Mtb*’s evolutionary lead, the science will have to shift beyond its present course of ‘activation of pathways’ to a systems-level, more subtle comprehension of host and pathogen behaviour. It is only then that we can really release the promise of autophagy-directed host therapies against TB.

### 6.3 Controversies, limitations, and unresolved questions

Beyond the strategic blind spots discussed above, several more fundamental questions remain open and are unlikely to be resolved by incremental compound discovery alone.

First, autophagy’s role in TB is not uniformly protective: it can support bacterial clearance, but when dysregulated, it can also contribute to excessive inflammation and tissue damage (Pentimalli, 2019). What determines which outcome dominates in a given patient, lesion, or stage of infection is not currently known, and this review treats it as an open mechanistic question rather than a caveat to be noted once and set aside.

Second, the mTORC1-inhibition paradox described in Section 4.1 is not a minor exception to an otherwise settled picture. If inhibiting a central upstream repressor of autophagy can, in some contexts, favour rather than restrict *Mtb* proliferation (Patel et al., 2024), this challenges the premise — implicit throughout much of the preclinical literature — that autophagy induction is uniformly beneficial. This paradox has not been mechanistically resolved and warrants dedicated investigation rather than being absorbed into a general statement about mTOR inhibitor pharmacology. Third, the pharmacological tools most commonly used to study autophagy in TB, including chloroquine, lack specificity for the autophagy pathway (Section 5). Because these agents act on multiple cellular processes simultaneously, a substantial fraction of the “autophagy-dependent” antimycobacterial efficacy reported in the preclinical literature has not been rigorously dissociated from off-target activity, and the true magnitude of autophagy’s independent contribution to bacterial clearance in these studies remains uncertain.

Fourth, as discussed in Section 6.2, the evidence base for autophagy-targeted HDT in TB is drawn almost entirely from immortalised cell lines and murine infection models. Given known interspecies differences in TLR signaling, IRGM function, and lysosomal biology between mice and humans, it is genuinely unresolved how much of this preclinical literature will translate to human granuloma biology, and this uncertainty applies across essentially every compound class discussed in this review, not to any single agent. Finally, despite more than two decades of preclinical interest in autophagy as an HDT target for TB, no randomised controlled trial has yet demonstrated a clear clinical benefit specifically attributable to autophagy induction, as distinct from the broader immunomodulatory or antimycobacterial effects of the repurposed drugs tested (Section 5). The fact that there is still such a gap between mechanistic approach and clinical studies is itself a challenge in its own right, and should not be overlooked as something that time alone will solve through more preclinical studies.

## 7 Conclusion

In conclusion, autophagy has been identified as a cellular process that can be therapeutically exploited with host-directed therapies (HDTs) to combat *Mtb*, particularly in the context of increasing drug resistance. This review discusses the prominent pathways *Mtb* exploits to subvert autophagy and the promise of pharmacological and repurposed compounds to overcome this inhibition and improve bacterial clearance. Although promising preclinical results have been shown, enormous hurdles must be overcome to translate autophagy modulators into effective clinical therapies. These include the lack of specific biomarkers, off-target effects, and poor human model systems. To address these gaps, there needs to be more targeted drug design, improved modelling of host-pathogen interactions, and integrated validation in the clinic. Ultimately, to exploit the full potential of autophagy-based host-directed therapy for tuberculosis control, a detailed, systems-level understanding of *Mtb*–host interactions will be necessary.
