# Supplementary material for: Autophagy in tuberculosis: advances, translational gaps, and the promise of host-directed therapies
Source: Front Cell Infect Microbiol. 2026 Aug 24;16:1896798. doi: 10.3389/fcimb.2026.1896798 (PMC13548514; doi:10.3389/fcimb.2026.1896798)
Supplement: Supplementary file 1 [file DataSheet1.docx]

**Autophagy in Tuberculosis: Advances, Translational Gaps, and the Promise of Host-Directed Therapies**

**Supplementary References (List appearing in Supplementary Table 1)**

Bai, X., Oberley-Deegan, R. E., Bai, A., Ovrutsky, A. R., Kinney, W. H., Weaver, M., Zhang, G., Honda, J. R., & Chan, E. D. (2016). Curcumin enhances human macrophage control of Mycobacterium tuberculosis infection. *Respirology*, *21*(5), 951-957. <https://doi.org/10.1111/resp.12762>

Barrientos, O. M., Juárez, E., Gonzalez, Y., Castro‐Villeda, D. A., Torres, M., & Guzmán‐Beltrán, S. (2021). Loperamide exerts a direct bactericidal effect against M. tuberculosis, M. bovis, M. terrae and M. smegmatis. *Letters in Applied Microbiology*, *72*(3), 351-356. <https://doi.org/10.1111/lam.13432>

Bhatt, K., Bhagavathula, M., Verma, S., Timmins, G. S., Deretic, V. P., Ellner, J. J., & Salgame, P. (2021). Rapamycin modulates pulmonary pathology in a murine model of Mycobacterium tuberculosis infection. *Dis Model Mech*, *14*(10). <https://doi.org/10.1242/dmm.049018>

Bruiners, N., Dutta, N. K., Guerrini, V., Salamon, H., Yamaguchi, K. D., Karakousis, P. C., & Gennaro, M. L. (2020). The anti-tubercular activity of simvastatin is mediated by cholesterol-dependent regulation of autophagy via the AMPK-mTORC1-TFEB axis. *bioRxiv*, 2020.2003.2004.977579. <https://doi.org/10.1101/2020.03.04.977579>

Cerni, S., Shafer, D., To, K., & Venketaraman, V. (2019). Investigating the Role of Everolimus in mTOR Inhibition and Autophagy Promotion as a Potential Host-Directed Therapeutic Target in Mycobacterium tuberculosis Infection. *J Clin Med*, *8*(2). <https://doi.org/10.3390/jcm8020232>

Chandra, V., Bhagyaraj, E., Nanduri, R., Ahuja, N., & Gupta, P. (2015). NR1D1 ameliorates Mycobacterium tuberculosis clearance through regulation of autophagy. *Autophagy*, *11*(11), 1987-1997. <https://doi.org/10.1080/15548627.2015.1091140>

Chen, C., Gardete, S., Jansen, R. S., Shetty, A., Dick, T., Rhee, K. Y., & Dartois, V. (2018). Verapamil Targets Membrane Energetics in Mycobacterium tuberculosis. *Antimicrob Agents Chemother*, *62*(5). <https://doi.org/10.1128/aac.02107-17>

Cheng, C. Y., Gutierrez, N. M., Marzuki, M. B., Lu, X., Foreman, T. W., Paleja, B., Lee, B., Balachander, A., Chen, J., Tsenova, L., Kurepina, N., Teng, K. W. W., West, K., Mehra, S., Zolezzi, F., Poidinger, M., Kreiswirth, B., Kaushal, D., Kornfeld, H.,...Singhal, A. (2017). Host sirtuin 1 regulates mycobacterial immunopathogenesis and represents a therapeutic target against tuberculosis. *Sci Immunol*, *2*(9). <https://doi.org/10.1126/sciimmunol.aaj1789>

Choi, S. W., Gu, Y., Peters, R. S., Salgame, P., Ellner, J. J., Timmins, G. S., & Deretic, V. (2018). Ambroxol Induces Autophagy and Potentiates Rifampin Antimycobacterial Activity. *Antimicrob Agents Chemother*, *62*(9). <https://doi.org/10.1128/aac.01019-18>

Cox, D. J., Coleman, A. M., Gogan, K. M., Phelan, J. J., C, Ó. M., Dunne, P. J., Basdeo, S. A., & Keane, J. (2020). Inhibiting Histone Deacetylases in Human Macrophages Promotes Glycolysis, IL-1β, and T Helper Cell Responses to Mycobacterium tuberculosis. *Front Immunol*, *11*, 1609. <https://doi.org/10.3389/fimmu.2020.01609>

Dutta, N. K., Bruiners, N., Zimmerman, M. D., Tan, S., Dartois, V., Gennaro, M. L., & Karakousis, P. C. (2020). Adjunctive Host-Directed Therapy With Statins Improves Tuberculosis-Related Outcomes in Mice. *J Infect Dis*, *221*(7), 1079-1087. <https://doi.org/10.1093/infdis/jiz517>

Fang, F., Ge, Q., Li, R., Lv, J., Zhang, Y., Feng, A., Kelly, G. T., Wang, H., Wang, X., Song, C., Wang, T., & Qian, Z. (2020). LPS restores protective immunity in macrophages against Mycobacterium tuberculosis via autophagy. *Mol Immunol*, *124*, 18-24. <https://doi.org/10.1016/j.molimm.2020.05.001>

Giraud-Gatineau, A., Coya, J. M., Maure, A., Biton, A., Thomson, M., Bernard, E. M., Marrec, J., Gutierrez, M. G., Larrouy-Maumus, G., Brosch, R., Gicquel, B., & Tailleux, L. (2020). The antibiotic bedaquiline activates host macrophage innate immune resistance to bacterial infection. *Elife*, *9*. <https://doi.org/10.7554/eLife.55692>

Gonzalo, X., Bielecka, M. K., Tezera, L., Elkington, P., & Drobniewski, F. (2022). Anti-Tuberculosis Activity of Three Carbapenems, Clofazimine and Nitazoxanide Using a Novel Ex Vivo Phenotypic Drug Susceptibility Model of Human Tuberculosis. *Antibiotics (Basel)*, *11*(10). <https://doi.org/10.3390/antibiotics11101274>

Gupta, P. K., Jahagirdar, P., Tripathi, D., Devarajan, P. V., & Kulkarni, S. (2023). Macrophage targeted polymeric curcumin nanoparticles limit intracellular survival of Mycobacterium tuberculosis through induction of autophagy and augment anti-TB activity of isoniazid in RAW 264.7 macrophages. *Front Immunol*, *14*, 1233630. <https://doi.org/10.3389/fimmu.2023.1233630>

He, F., Wu, X., Zhang, Q., Li, Y., Ye, Y., Li, P., Chen, S., Peng, Y., Hardeland, R., & Xia, Y. (2021). Bacteriostatic Potential of Melatonin: Therapeutic Standing and Mechanistic Insights \[Review\]. *Frontiers in Immunology*, *12*. <https://doi.org/10.3389/fimmu.2021.683879>

Hwang, H. Y., Shim, J. S., Kim, D., & Kwon, H. J. (2021). Antidepressant drug sertraline modulates AMPK-MTOR signaling-mediated autophagy via targeting mitochondrial VDAC1 protein. *Autophagy*, *17*(10), 2783-2799. <https://doi.org/10.1080/15548627.2020.1841953>

Jasim, T. M., Alabbassi, M., Almuqdadi, S., & Kamel, J. (2017). Anti-bacterial Properties of Melatonin against Mycobacterium Tuberculosis in Vitro. *Iraqi Journal of Pharmaceutical Sciences ( P-ISSN: 1683 - 3597 , E-ISSN : 2521 - 3512)*, *19*, 59-63. <https://doi.org/10.31351/vol19iss2pp59-63>

Juárez, E., Carranza, C., Sánchez, G., González, M., Chávez, J., Sarabia, C., Torres, M., & Sada, E. (2016). Loperamide Restricts Intracellular Growth of Mycobacterium tuberculosis in Lung Macrophages. *Am J Respir Cell Mol Biol*, *55*(6), 837-847. <https://doi.org/10.1165/rcmb.2015-0383OC>

Kim, J. K., Park, E. J., & Jo, E. K. (2022). Itaconate, Arginine, and Gamma-Aminobutyric Acid: A Host Metabolite Triad Protective Against Mycobacterial Infection. *Front Immunol*, *13*, 832015. <https://doi.org/10.3389/fimmu.2022.832015>

Kim, Y. S., Lee, H. M., Kim, J. K., Yang, C. S., Kim, T. S., Jung, M., Jin, H. S., Kim, S., Jang, J., Oh, G. T., Kim, J. M., & Jo, E. K. (2017). PPAR-α Activation Mediates Innate Host Defense through Induction of TFEB and Lipid Catabolism. *J Immunol*, *198*(8), 3283-3295. <https://doi.org/10.4049/jimmunol.1601920>

Lam, K. K. Y., Zheng, X., Forestieri, R., Balgi, A. D., Nodwell, M., Vollett, S., Anderson, H. J., Andersen, R. J., Av-Gay, Y., & Roberge, M. (2012). Nitazoxanide Stimulates Autophagy and Inhibits mTORC1 Signaling and Intracellular Proliferation of Mycobacterium tuberculosis. *PLOS Pathogens*, *8*(5), e1002691. <https://doi.org/10.1371/journal.ppat.1002691>

Lee, H. J., Kang, S. J., Woo, Y., Hahn, T. W., Ko, H. J., & Jung, Y. J. (2020). TLR7 Stimulation With Imiquimod Induces Selective Autophagy and Controls Mycobacterium tuberculosis Growth in Mouse Macrophages. *Front Microbiol*, *11*, 1684. <https://doi.org/10.3389/fmicb.2020.01684>

Liu, H., Li, S., Deng, L., Shi, Z., Jiang, C., Shu, J., Liu, Y., Deng, X., Wang, J., Guo, Z., & Qiu, J. (2024). Repurposing Loperamide as an Anti-Infection Drug for the Treatment of Intracellular Bacterial Pathogens. *Engineering*, *39*, 180-193. <https://doi.org/https://doi.org/10.1016/j.eng.2024.01.011>

Maiga, M., Ahidjo, B. A., Maiga, M. C., Cheung, L., Pelly, S., Lun, S., Bougoudogo, F., & Bishai, W. R. (2015). Efficacy of Adjunctive Tofacitinib Therapy in Mouse Models of Tuberculosis. *EBioMedicine*, *2*(8), 868-873. <https://doi.org/10.1016/j.ebiom.2015.07.014>

Mishra, R., Krishan, S., Rai, P. K., Kapur, P., Khayyam, K. U., Azharuddin, M., Sharma, K., & Sharma, M. (2022). Effect and possible mechanisms of metformin as adjuvant therapy in the management of tuberculosis: A prospective study. *Clinical Epidemiology and Global Health*, *16*, 101106. <https://doi.org/https://doi.org/10.1016/j.cegh.2022.101106>

Nasiri, M. J., Lutfy, K., & Venketaraman, V. (2024). Challenges of Multidrug-Resistant Tuberculosis Meningitis: Current Treatments and the Role of Glutathione as an Adjunct Therapy. *Vaccines (Basel)*, *12*(12). <https://doi.org/10.3390/vaccines12121397>

Nieto-Patlán, E., Serafín-López, J., Wong-Baeza, I., Pérez-Tapia, S. M., Cobos-Marín, L., Estrada-Parra, S., Estrada-García, I., Chávez-Blanco, A. D., & Chacón-Salinas, R. (2019). Valproic acid promotes a decrease in mycobacterial survival by enhancing nitric oxide production in macrophages stimulated with IFN-γ. *Tuberculosis*, *114*, 123-126. <https://doi.org/https://doi.org/10.1016/j.tube.2018.12.007>

Niu, Y., Wu, Z., Hu, Q., Wu, Y., Jiang, Q., & Yang, X. (2025). Discovery of acetohydroxyacid synthase inhibitors as anti-tuberculosis lead compounds from natural products. *Bioorganic & Medicinal Chemistry*, *118*, 118041. <https://doi.org/https://doi.org/10.1016/j.bmc.2024.118041>

Novita, B. D., Christy Mulyono, A., & Erwin, F. (2021). Metformin for Tuberculosis Infection. In J. Akhtar, U. Ahmad, Badruddeen, & M. I. Khan (Eds.), *Metformin - Pharmacology and Drug Interactions*. IntechOpen. <https://doi.org/10.5772/intechopen.99794>

Padmapriyadarsini, C., Szumowski, J. D., Akbar, N., Shanmugasundaram, P., Jain, A., Bathragiri, M., Pattnaik, M., Turuk, J., Karunaianantham, R., Balakrishnan, S., Pati, S., Kumar, A. K. H., Rathore, M. K., Raja, J., Naidu, K. R., Horn, J., Whitworth, L., Sewell, R., Ramakrishnan, L.,...Edelstein, P. H. (2024). A Dose-Finding Study to Guide Use of Verapamil as an Adjunctive Therapy in Tuberculosis. *Clin Pharmacol Ther*, *115*(2), 324-332. <https://doi.org/10.1002/cpt.3108>

Rao, M., Valentini, D., Zumla, A., & Maeurer, M. (2018). Evaluation of the efficacy of valproic acid and suberoylanilide hydroxamic acid (vorinostat) in enhancing the effects of first-line tuberculosis drugs against intracellular Mycobacterium tuberculosis. *Int J Infect Dis*, *69*, 78-84. <https://doi.org/10.1016/j.ijid.2018.02.021>

Schiebler, M., Brown, K., Hegyi, K., Newton, S. M., Renna, M., Hepburn, L., Klapholz, C., Coulter, S., Obregón-Henao, A., Henao Tamayo, M., Basaraba, R., Kampmann, B., Henry, K. M., Burgon, J., Renshaw, S. A., Fleming, A., Kay, R. R., Anderson, K. E., Hawkins, P. T.,...Floto, R. A. (2015). Functional drug screening reveals anticonvulsants as enhancers of mTOR-independent autophagic killing of Mycobacterium tuberculosis through inositol depletion. *EMBO Mol Med*, *7*(2), 127-139. <https://doi.org/10.15252/emmm.201404137>

Shankaran, D., Singh, A., Dawa, S., Arumugam, P., Gandotra, S., & Rao, V. (2023). The antidepressant sertraline provides a novel host directed therapy module for augmenting TB therapy. *Elife*, *12*. <https://doi.org/10.7554/eLife.64834>

Sharma, V., Makhdoomi, M., Singh, L., Kumar, P., Khan, N., Singh, S., Verma, H. N., Luthra, K., Sarkar, S., & Kumar, D. (2021). Trehalose limits opportunistic mycobacterial survival during HIV co-infection by reversing HIV-mediated autophagy block. *Autophagy*, *17*(2), 476-495. <https://doi.org/10.1080/15548627.2020.1725374>

Shukla, H., Kumar, V., Singh, A. K., Rastogi, S., Khan, S. R., Siddiqi, M. I., Krishnan, M. Y., & Akhtar, M. S. (2015). Isocitrate lyase of Mycobacterium tuberculosis is inhibited by quercetin through binding at N-terminus. *Int J Biol Macromol*, *78*, 137-141. <https://doi.org/10.1016/j.ijbiomac.2015.04.005>

Singh, P., & Subbian, S. (2018). Harnessing the mTOR Pathway for Tuberculosis Treatment. *Front Microbiol*, *9*, 70. <https://doi.org/10.3389/fmicb.2018.00070>

Singhal, A., Jie, L., Kumar, P., Hong, G. S., Leow, M. K.-S., Paleja, B., Tsenova, L., Kurepina, N., Chen, J., Zolezzi, F., Kreiswirth, B., Poidinger, M., Chee, C., Kaplan, G., Wang, Y. T., & De Libero, G. (2014). Metformin as adjunct antituberculosis therapy. *Science Translational Medicine*, *6*(263), 263ra159-263ra159. <https://doi.org/doi:10.1126/scitranslmed.3009885>

Stanley, S. A., Barczak, A. K., Silvis, M. R., Luo, S. S., Sogi, K., Vokes, M., Bray, M. A., Carpenter, A. E., Moore, C. B., Siddiqi, N., Rubin, E. J., & Hung, D. T. (2014). Identification of host-targeted small molecules that restrict intracellular Mycobacterium tuberculosis growth. *PLoS Pathog*, *10*(2), e1003946. <https://doi.org/10.1371/journal.ppat.1003946>

Sundaramurthy, V., Barsacchi, R., Samusik, N., Marsico, G., Gilleron, J., Kalaidzidis, I., Meyenhofer, F., Bickle, M., Kalaidzidis, Y., & Zerial, M. (2013). Integration of Chemical and RNAi Multiparametric Profiles Identifies Triggers of Intracellular Mycobacterial Killing. *Cell host & microbe*, *13*(2), 129-142. <https://doi.org/https://doi.org/10.1016/j.chom.2013.01.008>

Swain, S. S., Rout, S. S., Sahoo, A., Oyedemi, S. O., & Hussain, T. (2021). Antituberculosis, antioxidant and cytotoxicity profiles of quercetin: a systematic and cost-effective in silico and in vitro approach. *Natural Product Research*, *36*(18), 4757-4761. <https://doi.org/10.1080/14786419.2021.2008387>

Ullah, O., Hanif, N., Ayesha, Mufti, A. Q., Amjad, F., Manzoor, M., Jameel, E., & Fatima, S. (2024). Insilico Insights into Resveratrol as a Potential Inhibitor of Mycobacterium Tuberculosis Enoyl-ACP Reductase (InhA) Protein: Insilico Insights into Mycobacterium Tuberculosis Enoyl-ACP Reductase. *Futuristic Biotechnology*, *4*(03), 27-33. <https://doi.org/10.54393/fbt.v4i03.134>

Wang, J., Sha, J., Strong, E., Chopra, A. K., & Lee, S. (2022). FDA-Approved Amoxapine Effectively Promotes Macrophage Control of Mycobacteria by Inducing Autophagy. *Microbiol Spectr*, *10*(5), e0250922. <https://doi.org/10.1128/spectrum.02509-22>

Yang, C. S., Kim, J. J., Lee, H. M., Jin, H. S., Lee, S. H., Park, J. H., Kim, S. J., Kim, J. M., Han, Y. M., Lee, M. S., Kweon, G. R., Shong, M., & Jo, E. K. (2014). The AMPK-PPARGC1A pathway is required for antimicrobial host defense through activation of autophagy. *Autophagy*, *10*(5), 785-802. <https://doi.org/10.4161/auto.28072>

Zhang, Q., Sun, J., Wang, Y., He, W., Wang, L., Zheng, Y., Wu, J., Zhang, Y., & Jiang, X. (2017). Antimycobacterial and Anti-inflammatory Mechanisms of Baicalin via Induced Autophagy in Macrophages Infected with Mycobacterium tuberculosis. *Front Microbiol*, *8*, 2142. <https://doi.org/10.3389/fmicb.2017.02142>
